# Orientin Induces G0/G1 Cell Cycle Arrest and Mitochondria Mediated Intrinsic Apoptosis in Human Colorectal Carcinoma HT29 Cells

**DOI:** 10.3390/biom9090418

**Published:** 2019-08-27

**Authors:** Kalaiyarasu Thangaraj, Balamuralikrishnan Balasubramanian, Sungkwon Park, Karthi Natesan, Wenchao Liu, Vaiyapuri Manju

**Affiliations:** 1 Department of Microbiology and Biotechnology, Bharath Institute of Higher Education and Research, Tamilnadu 600045, India; 2 Molecular Oncology Lab, Department of Biochemistry, Periyar University, Tamilnadu 636011, India; 3 Department of Food Science and Biotechnology, College of Life Science, Sejong University, Seoul 05006, Korea; 4 Genomic Division, National Academy of Agricultural Science, RDA, Jeollabuk 560500, Korea; 5 Department of Animal Science, College of Agriculture, Guangdong Ocean University, Zhanjiang 524088, China

**Keywords:** colorectal cancer, orientin, cell cycle arrest, Bcl-2 family proteins, apoptosis

## Abstract

Colorectal carcinoma is one of the utmost diagnosed cancer with a steep increase in mortality rate. The incidence has been increasing in developing countries like India due to a westernization life style. Flavonoids have been explored widely for its various pharmacological activity including antitumor activity. Orientin, an analogue of luteolin (citrus flavonoid) isolated from rooibos and tulsi leaves is also expected to deliver significant antitumor activity similar to that of luteolin. The present study anticipates exploring the antitumor activity of orientin against colorectal carcinoma cells (HT29). Orientin exhibited remarkable cytotoxicity and antiproliferative activity against HT29 cells, which is clearly evident from tetrazolium based cytotoxicity and lactate dehydrogenase release assays. Orientin induce G0/G1 cell cycle arrest and regulates cyclin and cyclin-dependent protein kinases in order to prevent the entry of the cell cycle to the S phase. Annexin V-FITC (V-Fluorescein Isothiocyanate) dual staining reveals the apoptotic induction ability of orientin. The Bcl-2 family proteins along with the inhibitor of apoptotic proteins were regulated and the tumor suppressor p-53 expression have been decreased. In conclusion, our results proposed that orientin could be a potent chemotherapeutic agent against colorectal cancer after ascertaining their molecular mechanisms.

## 1. Introduction

Colorectal cancer (CRC) is the third most recurrently diagnosed cancer and fourth most likely cause of cancer mortality worldwide with increasing incidence in recent years [1]. The incidence of CRC accounts for more than 8% of total cancer incidence among the cancers that occurs in both men and women [2]. Despite the rapidity in drug development, antagonistic screening and promoted public health awareness, the global burden of CRC is anticipated to rise almost 60% by 2030 [3]. Therefore, much more effective therapeutic strategies are requisite in the present era in the treatment of CRC. In accordance with this, numerous reports have highlighted the role of flavonoids in inducing apoptotic signaling pathways and thereby thwarting the progression of CRC. A meta-analysis reveals that, the increase in consumption of few dietary flavonoids highly correlated with a decreased risk of colon and rectal cancer [4]. Flavonoids such as quercetin, luteolin, kaempferol, apigenin, epigallocatechin, hesperetin, naringenin and pelargonidin have been reported earlier to possess a protective effect against the development of recurrent adenomas and colon cancer [5]. The heterogeneous CRC involves multiple cascades of events accelerated by an array of oncogenes and tumor suppressor genes in the transformation of colonic epithelium into metastatic carcinoma [6]. The cell cycle includes an array of events that drives the proliferation of cells through a sequence of checkpoints that repairs DNA damage, genetic derangements and incomplete DNA replication. The cell cycle checkpoints protect transformed cells under genotoxic and replicative stress, thereby shielding the integrity of the genome [7]. The cell cycle progression involves the heterodimeric interaction of cyclins and cyclin-dependent protein kinases (CDK), which regulate the activities of target proteins by phosphorylation.

Reactive oxygen species (ROS), a heterogeneous group associates in the regulation of many cellular physiological and pathological processes. Mitochondrial respiration is the prime endogenous source of ROS and almost 90% of ROS are generated by mitochondria [8]. The enhanced production of intracellular ROS can pose a serious menace to cells by causing lipid peroxidation, protein oxidation, damage to nucleic acids and modulation of cellular functions such as proliferation, differentiation, growth and activation of programmed cell death pathway and ultimately leading to cell death. One of the possible mechanisms could be the intervention in the intracellular signaling pathways regulating cell survival and apoptosis [9]. Apoptosis plays a pivotal role in cellular proliferation, differentiation, senescence and death with typical characteristics of membrane blebbing, shrinkage of nucleus, condensation of chromatin, fragmentation of DNA and formation of apoptosis [10]. The programmed cell death process involving a highly complex cascade of cellular events occurs in either way, intrinsic and extrinsic depending upon the trigger of the death inducing signals [11]. Meanwhile, both signaling pathways induce apoptotic effector molecules to induce apoptosis. The apoptotic mechanism is orchestrated by the evolutionarily conserved B-cell leukemia/lymphoma 2 (Bcl-2) family proteins such as pro-apoptotic (e.g., Bax and Bak) and anti-apoptotic (e.g., Bcl-2 and Bcl-XL). Indeed, the ratio between Bcl-2 and Bax helps decide in part, the cell susceptibility to death inducing signals [12]. The pro-apoptotic proteins act in such a way that mitochondria permeabilizes, releases cytochrome C and subsequently activates the caspase cascade to trigger apoptosis mediated by mitochondria [13]. The death inducing ligands bind specifically to the cell surface receptors and instigate extrinsic apoptosis through intracellular activation of caspase-8 resulting in cell death [9]. Several reports have been demonstrated that the Bcl-2 and Bcl-XL are widely over expressed in different forms of cancers [14]. Therefore, the development of anticancer drugs to inhibit antiapoptotic proteins of Bcl-2 family serves to be a promising strategy for treating cancer [15].

Orientin (luteolin-8-C-glucoside), a water soluble glycosyl flavonoid rich in rooibos tea, isolated from *Ocimum sanctum*, *Phyllostachys* species, *Passiflora* species and *Trollius* species [16]. It exerts various pharmacological activities such as antioxidant, anti-inflammatory, neuroprotective, cardioprotective and antitumor effects [17,18]. Earlier studies reported that orientin exerts cytotoxicity in esophageal cancer EC109 cells [19] and MCF-7 breast cancer cells [20]. However, there is limited information on the effect of orientin against CRC in vitro and the putative mechanisms of cytotoxicity induced by orientin also remain unknown. The present study investigates the influence of orientin on proliferation, cell cycle arrest and apoptosis in human CRC cells (HT29) and to explore the underlying mechanisms involved in the pharmacological actions of orientin.

## 2. Materials and Methods

### 2.1. Materials and Reagents

Orientin, RPMI-1640 medium, DMSO, antibiotic antimycotic solution, trypsin–EDTA solution and MTT dye were procured from Sigma Chemicals, MO. FBS was purchased from Gibco-BRL, MD. The primary antibodies anti-Bcl-2 (#15071S), Bax (#2772S), Bcl-xL (#2764S), Bid (#2003S), procaspase-3 (#9662S), cleaved caspase-3 (#9661S), procaspase 9(#9508S), cleaved caspase 9 (#9509S), cytochrome C (#11940S), Smac/DIABLO (#2954S), AIF(#4642S), p21 (#2946S), p53 (#9282S), p-Rb (#9307S) or p-H2AX (#2577S) were procured from Cell Signaling Technology, MA. Anti-cyclin B1 (sc-245), CDK1 (sc-53219), CDC2 (sc-54), MDM2 (sc-965), PARP (sc-56196), cleaved PARP (sc-56196), X-linked inhibitor of apoptotic proteins (XIAP; sc-55550), survivin (sc-17779) or β-actin (sc-47778) antibodies, and HRP conjugated secondary antibodies (sc-2359) were purchased from Santa Cruz Biotechnology, CA. All further chemicals used in this study were of reagent or analytical grade and obtained from commercial suppliers.

### 2.2. Cell Culture Maintenance and Treatment

The HT29 cell lines were procured from National Center for Cell Sciences, India. Cells were cultured in RPMI-1640 medium supplemented with 10% FBS and 2-mM L-glutamine, 100 U/mL antibiotic antimycotic solution and maintained at 37 °C in CO_2_ (5%) incubator with 95% humidity. Orientin stock solution was prepared in DMSO (0.1%) and stored at −20 °C until use.

### 2.3. Tetrazolium Based Cell Viability Assay

The HT29 colon cells were treated with orientin and irinotecan (3.125 to 100 µM). Cell viability after 24 h was determined based on a MTT assay. Briefly, HT29 cells (3 × 10^3^ cells/well) were seeded in a 96-well plate and left overnight to get adhere. After removal of the medium, 200 µL of fresh medium added per well, containing 10 mmol/L HEPES (pH 7.4). 50 µL MTT was added and the plate was incubated for 2–4 h at 37 °C in the dark. After removal of spent medium, DMSO (200 µL) and Sorensen’s glycine buffer (25 µL) were added to the wells. The absorbance at 570 nm was determined using an ELISA plate reader (BioRad, Richmond, CA, USA). Meanwhile, the cytotoxicity of orientin on normal epithelial cells, Vero (normal kidney epithelial cell), was also evaluated.

### 2.4. Morphological Observation and Cell-Cycle Analysis

The HT29 cells (4 × 10^3^ cells/coverslip) were grown and treated (24 h) with orientin (3.125 to 100 µM) and further dissolved in methanol/acetic acid solution (3:1, *v*/*v*). The effect of orientin HT29 cells morphology was observed by bright-field inverted light microscope (400×). The cell-cycle distribution was analyzed by flow cytometry as described earlier [21].

### 2.5. Annexin V-FITC/PI Apoptotic Assay

Annexin V-fluorescein isothiocyanate/propidium iodide dual staining was employed to distinguish early and late apoptotic cells (BD Pharmingen, San Jose, CA, USA). Fluorescein isothiocyanate -conjugated Annexin V was used to quantify the loss of asymmetry of phosphatidylserine on cell membranes involved in apoptosis while propidium iodide differentiates the early apoptotic, the late apoptotic and necrotic cells [22]. Briefly, HT29 cells (3 × 10^5^ cells/well) were seeded in a 6-well plate and treated with the orientin for 48 h. Cells were gently washed twice with phosphate buffered saline accompanied by tryptic digestion (0.25% trypsin/EDTA) and washed yet again with PBS. All the cells including the floating and adherent ones were harvested, pooled and incubated with dual stain for 15 min on ice under dark. The cells were counted by a flow cytometer (Becton-Dickinson, San Jose, CA, USA).

### 2.6. Measurement of Intracellular ROS

The intracellular ROS was measured based on its conversion of 2′,7′-Dichlorodihydrofluorescein diacetate in to fluorescent DCFH. HT29 cells (1 × 10^5^ cells/mL) were seeded in black colored bottom plate with 96 wells and allowed to adhere overnight. After being treated with orientin (6.25, 12.5 and 25 µM), the cells were washed twice with PBS. Then the cells were incubated with 20 µM of DCFH-DA solution at 37 °C for 30 min and suspended in 200 µL of PBS. The qualitative analysis of ROS generation was carried out by a fluorescence microscope (Nikon Eclipse, Tokyo, Japan) with an excitation filter around 510–590 nm (40×) and estimated with Image J software (Version 1.45, NIH, Bethesda, MD, USA).

### 2.7. Western Blotting

Briefly, HT29 cells (1 × 10^6^ cells/mL) were treated for 24 h with Orientin (6.25, 12.5 and 25 µM). Cells were then washed with PBS and lysed using ice-cold RIPA (radio immuno precipitation assay) buffer. The separation of protein was carried out by subjecting samples on to 12% or 16% resolving polyacrylamide gels and electroblotting (25 mA) for 1 h at room temperature. After transferring the proteins to PVDF (polyvinylidene fluoride) membrane (Pierce, Rockford, IL, USA), the membranes were blocked for 1 h with a blocking buffer at room temperature. The primary antibodies namely anti-Bax (1:250), Bcl-2 (1:500), Bcl-xL (1:500), Bid (1:1000), procaspase-9 (1:1000), cleaved caspase-9 (1:1000), procaspase-3 (1:2000), cleaved caspase-3 (1:2000), mitochondrial cytochrome C, cytosolic cytochrome C, mitochondrial Smac/DIABLO (1:1000), cytosolic Smac/DIABLO, cyclin D1 (1:1000), cyclin E (1:1000), CDK2 (1:500), CDK4 (1:500), PARP (1:1000), cleaved PARP (1:1000), XIAP, Survivin, p-Rb (1:500), p53 (1:500), p-21^WAF1/CIP1^ (1:1000), p-H2AX (1:1000), COX-IV (1:1000) or β-actin (1:5000), were incubated at 4 °C overnight. HRP-conjugated polyclonal secondary antibody (1:3000) and Chemiluminescence Plus detection components were used.

### 2.8. Statistical Analysis

All the experimental data were evaluated using SPSS 16.0 and expressed as mean ± SD. One way ANOVA was carried out and the probability values of <0.0001, <0.01 and <0.05 were considered as significant.

## 3. Results

### 3.1. Orientin Exhibits Cytotoxicity in HT29 Cells

**Figure 1:**
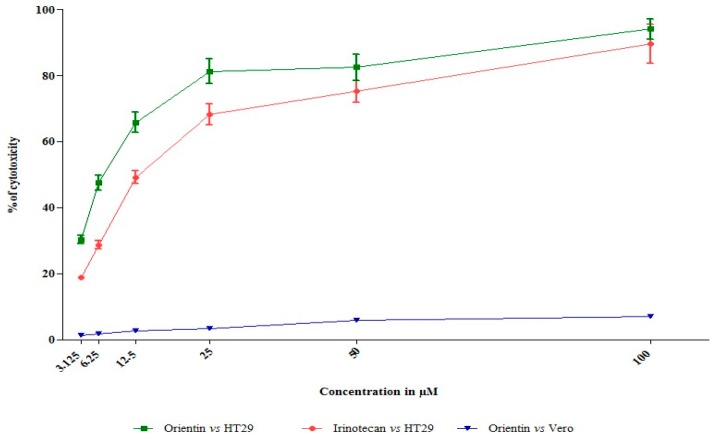
Effect of orientin on colorectal carcinoma and normal epithelial cell viability. Change in HT29 and normal epithelial cell viability with treatment of different concentrations of orientin and irinotecan were observed. The results were represented as mean ± SD of three independent parallel measurements.

Orientin and irinotecan (CPT-11) treated and untreated control HT29 cells were studied for determining the inhibition of cell viability using the tetrazolium based cytotoxicity assay. In addition, the cytotoxicity of orientin was also assessed in normal epithelial Vero cells. The percentage of viability was calculated at different concentrations (3.125 to 100 µM) for 24 h. Orientin exhibited potent dose dependent antiproliferative effect against HT29 cells as shown in Figure 1. Irinotecan (CPT-11) acting as the positive control demonstrated significant anti-proliferative potential against HT-29 cells. GI50 (50% growth inhibition) of orientin and irinotecan was found to be 12.55 and 5.19 µM, respectively, whereas in normal cells, no such cytotoxic effect was observed with the selected doses. The experimental findings revealed a potent inhibitory effect of orientin on carcinogenic HT29 cells without toxicity in normal epithelial Vero cells. The tetrazolium based antiproliferative study revealed that orientin significantly inhibits HT29 cell viability in a dose-dependent mode.

**Figure 2:**
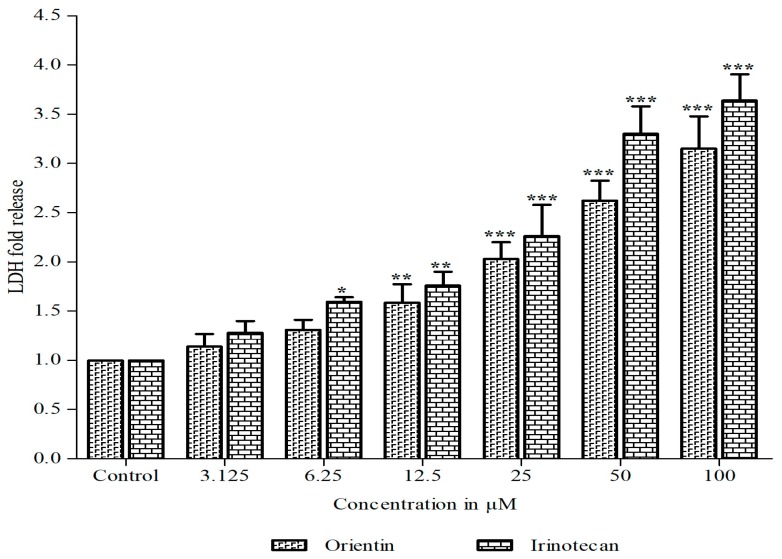
Effect of orientin on lactate dehydrogenase (LDH) release. The release of LDH in HT-29 cells treated with different concentration of orientin and irinotecan for 24 h. Results are expressed as the mean ± SD of three independent experiments. * *p* < 0.05, ** *p* < 0.01 and *** *p* < 0.001 vs. control.

In additional to quantify the extent of cellular toxicity in HT29 cells, a lactate dehydrogenase release assay was performed. Orientin significantly constrained the growth of HT29 cells and rendered cytotoxicity, which was clearly evident by the increasing amount of lactate dehydrogenase released in a dose dependent mode (Figure 2).

### 3.2. Morphological Changes Induced in HT29 Cells by Orientin

**Figure 3:**
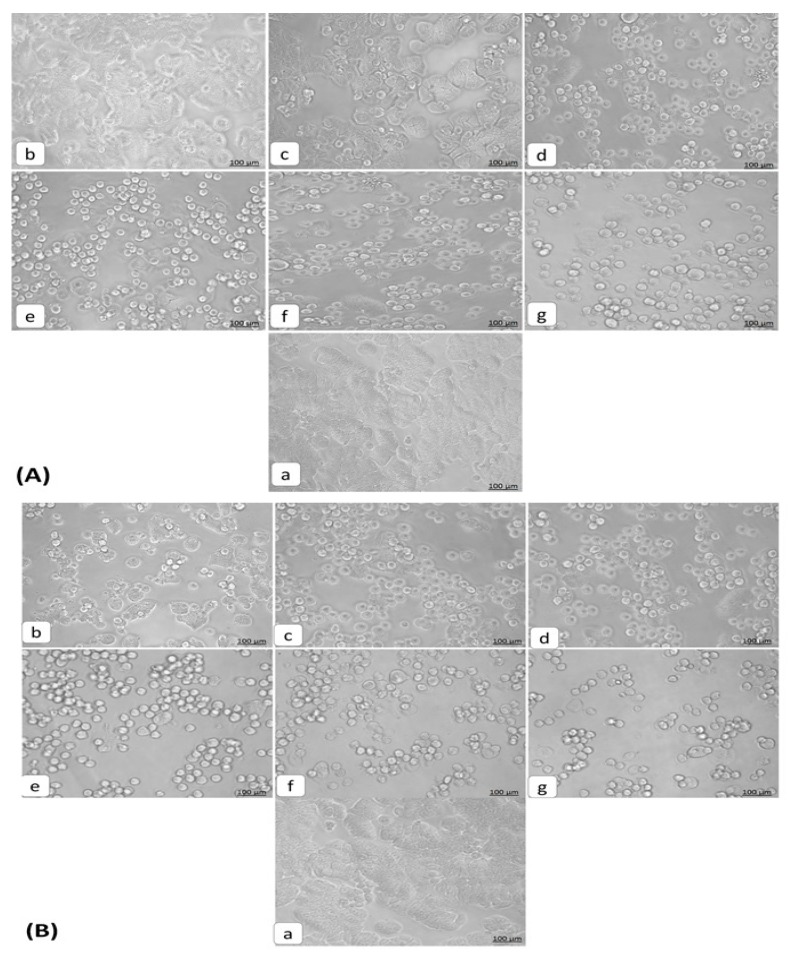
Morphological changes observed in HT29 cells on exposure to orientin and irinotecan. HT29 cells treated with different concentrations of orientin (**A**) and irinotecan (**B**) were observed after 24 h by inverted bright field microscopy (100 µm). (**a**) Untreated control, (**b**) 3.125 µM, (**c**) 6.25 µM, (**d**) 12.5 µM, (**e**) 25 µM, (**f**) 50 µM and (**g**) 100 µM. The treated cells were found to be shrunken, rounded off and detached from the layer.

The morphology of HT29 cells treated with orientin (3.125–100 µM) was observed by inverted light microscopy. The untreated cells were found to be healthy and polyhedral in shape with a distinct cytoskeleton. Orientin treated cell loss their normal architecture, found to be rounded and shrunken in nature. The increased number of detached cells with the increasing concentration reveals the apoptotic effect of orientin. The light microscopic observations showed typical variations in cell morphology after 24 h exposure (Figure 3). This includes the cell shrinkage from its polyhedral origin, rounded off, membrane blebbing and detachment of cells making substantiation for apoptosis [21]. The overall findings of cytotoxic assays and morphological observations obviously state that orientin exerted a significant antiproliferative effect against HT29 cells dose dependently. Thus, based on the cytotoxicity and morphological observations, further experiments were carried out at 12.5 µM (GI50) and 6.25 µM (sub GI50) concentrations of orientin and compared with 5 µM (GI50) of standard irinotecan.

### 3.3. Initiation of the G0/G1 Phase Cell Cycle Arrest by Orientin

**Figure 4:**
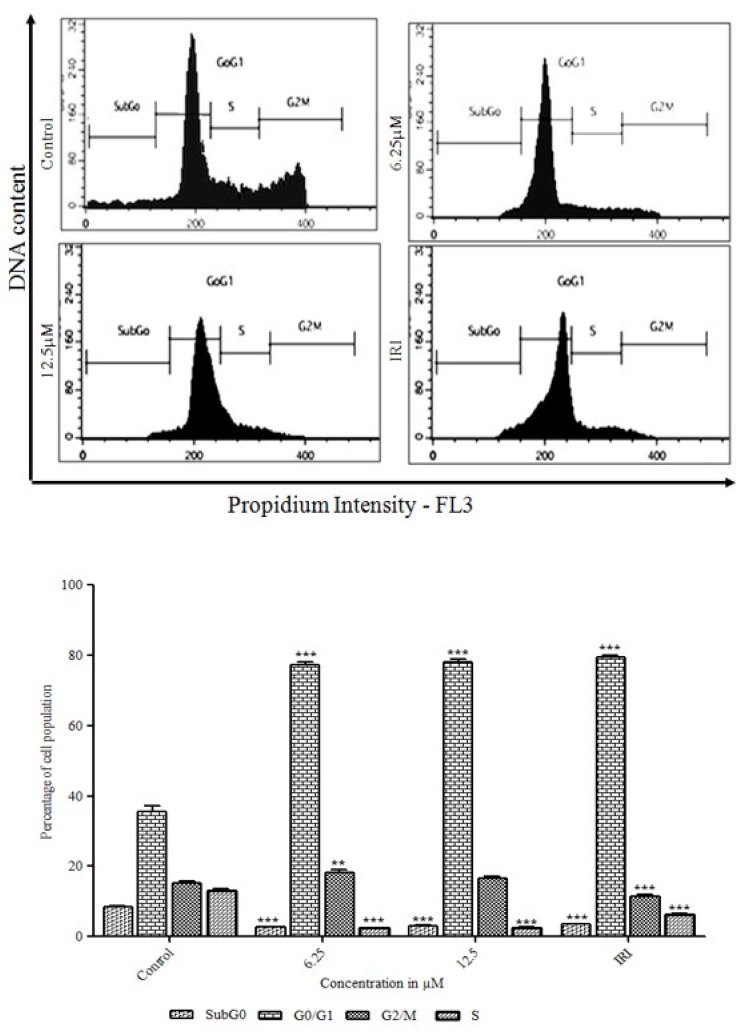
Effect of orientin on cell cycle phase distribution in HT29 cells. HT-29 cells were treated with different concentrations of orientin and analyzed after 24 h for DNA content by flow cytometry. Histogram representing the PI (propidium iodide) staining (FL2-A) of orientin treated cells. The data shown are representative of three independent experiments with similar findings. The significant differences from control were indicated by * *p* < 0.05, ** *p* < 0.01 and *** *p* < 0.0001 (considered as statistically significant).

The molecular mechanism of orientin induced anti-proliferation in HT29 cells was determined by their effects on cell cycle progression using PI. Flow cytometry analyses of cell cycle distribution in (6 µM and 12.5 µM) orientin and irinotecan treated cells were illustrated in Figure 4. Orientin was shown to block the cell progression at G0/G1 phase itself. After treatment with orientin, a substantial increase in G0/G1 phase population was observed when compared with control cells. Our data revealed that the percentage of untreated cells in the sub-G1, G0/G1, S and G2/M phases were determined to be 8.39%, 35.56%, 15.31% and 13.03%, respectively; whereas GI50 of orientin treated cells were 3.03%, 78.21%, 16.49% and 2.55%, respectively (Figure 4). The results suggest that orientin arrest at G0/G1 phase and the cell population was increased and this could be responsible for the anti-proliferation of HT29 cells.

### 3.4. Orientin Induced p21^WAF1/CIP1^ Mediated G0/G1 Arrest in HT29 Cells

**Figure 5:**
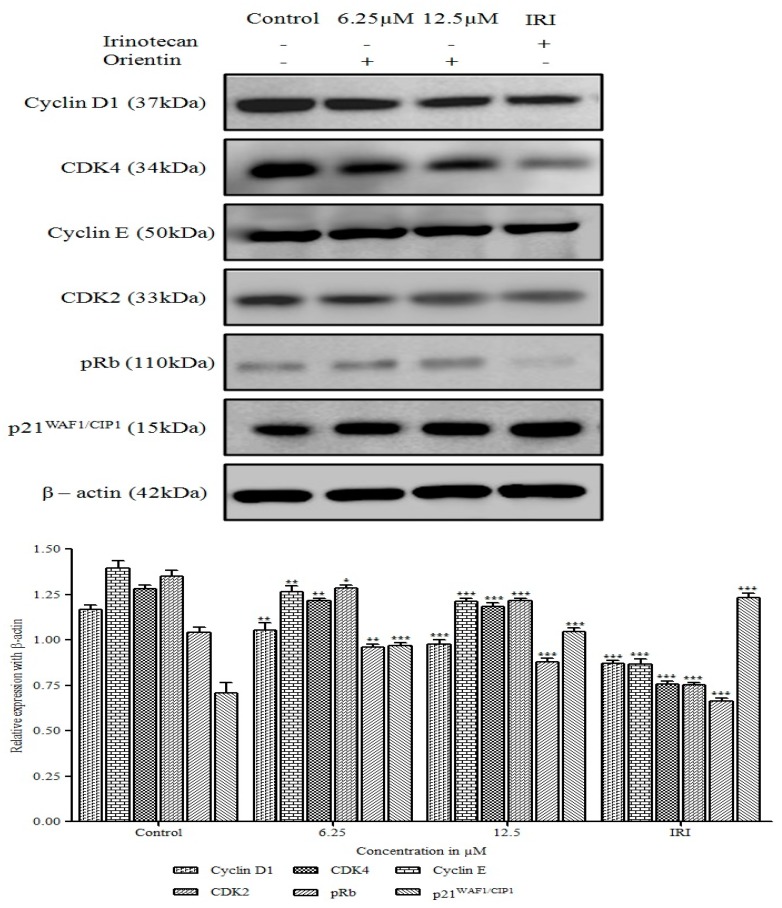
Effect of orientin on cell cycle regulatory proteins. HT29 cells were treated with orientin (6.25 and 12.5 µM) and irinotecan for 24 h. The immunoblot analysis of cyclins, CDKs and their inhibitors involved in G0/G1 phase were carried out. Orientin reduced the expression of cyclins, pRb and CDKs, in contrast, increased the level of p21^WAF1/CIP1^. β-actin was used as an internal control. Quantitative expression of proteins shown after normalization to β-actin. The data presented are the mean ± SD of results from three independent experiments (* *p* < 0.05, ** *p* < 0.01 and *** *p* < 0.001 vs. control).

To determine the probable mechanism underlying the cell cycle arrest by orientin in HT29 cells, the change in the regulatory proteins of cell cycle such as cyclins (D1 and E), CDK (CDK2 and CDK4), p21^WAF1/CIP1^ and p-Rb at the G0/G1 phase was observed using an immunoblotting analysis. As shown in Figure 5, orientin significantly (* *p* < 0.05, ** *p* < 0.01 and *** *p* < 0.001) decreased the expression of cyclins, CDK and concomitantly increased the level of cyclin D-CDK complexes inhibitor, p21^WAF1/CIP1^ dose dependently. The decreased level of cyclin D-CDK2 and cyclin E-CDK-4 complexes made evident orientin induced cell cycle arrest at G0/G1 phase in HT-29 cells. p-Rb, the primary target of the G1 kinases was also found to be significantly decreased dose dependently upon treatment with orientin (** *p* < 0.01 and *** *p* < 0.001).

To determine the probable mechanism underlying the cell cycle arrest by orientin in HT29 cells, the change in the regulatory proteins of cell cycle such as cyclins (D1 and E), CDK (CDK2 and CDK4), p21^WAF1/CIP1^ and p-Rb at the G0/G1 phase was observed using an immunoblotting analysis. As shown in Figure 5, orientin significantly (* *p* < 0.05, ** *p* < 0.01 and *** *p* < 0.001) decreased the expression of cyclins, CDK and concomitantly increased the level of cyclin D-CDK complexes inhibitor, p21^WAF1/CIP1^ dose dependently. The decreased level of cyclin D-CDK2 and cyclin E-CDK-4 complexes made evident orientin induced cell cycle arrest at the G0/G1 phase in HT-29 cells. p-Rb, the primary target of the G1 kinases was also found to be significantly decreased dose dependently upon treatment with orientin (** *p* < 0.01 and *** *p* < 0.001).

### 3.5. Orientin Induces Apoptosis in HT29 Cells

**Figure 6:**
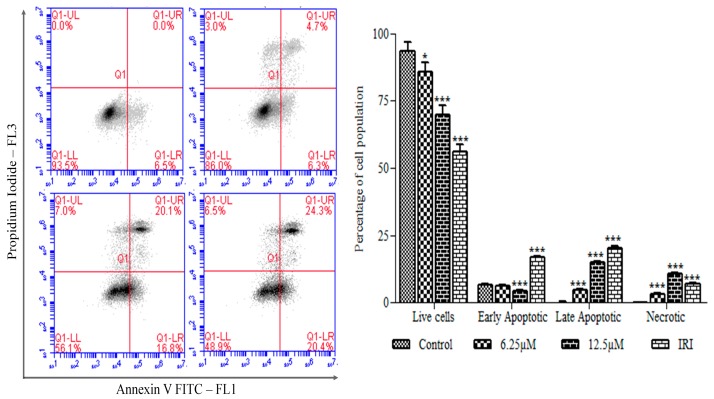
Effect of orientin on apoptosis in HT29 cells. Cells were treated with 6.25 and 12.5 μM orientin and irinotecan for 24 h, and the induction of cell apoptosis was examined by flow cytometry. LR represents early apoptotic cells and UR represents late apoptotic cells. Percentage of early and late apoptotic cells is shown in the bar graph. Data are shown as mean ± SD of three parallel independent experiments. * *p* < 0.05 and *** *p* < 0.001 vs. control.

To further substantiate that their anti-proliferative activities occur due to apoptosis, the quantitative assessment of apoptosis was determined by Annexin V-FITC/PI staining by flow cytometry (Figure 6). HT29 cells treated with orientin for 24 h were stained with Annexin-V and PI consecutively. The live cell population and the cells undergoing early apoptosis (Annexin^+^/PI^−^), late apoptosis (Annexin^+^/PI^+^) and necrosis (Annexin^−^/PI^+^) were quantified. The findings revealed that orientin treated HT29 cells underwent apoptosis and showed a significant (*p* < 0.05) decrease in the proportion of live cells and enhanced the translocation of phosphatidyl serine (Annexin V positive cells) dose dependently. The percentage of cells undergoing early and late apoptosis on exposure to orientin at 6 and 12.5 µM were found to be 11%, and 36.9%, respectively, which is comparable to that of irinotecan (44.7%). However, the level of cells undergoing necrosis was limited making obvious that orientin exhibited an anti-proliferative effect with the increasing rate of apoptosis.

### 3.6. Intracellular Accumulation of ROS by Orientin in HT29 Cells

**Figure 7:**
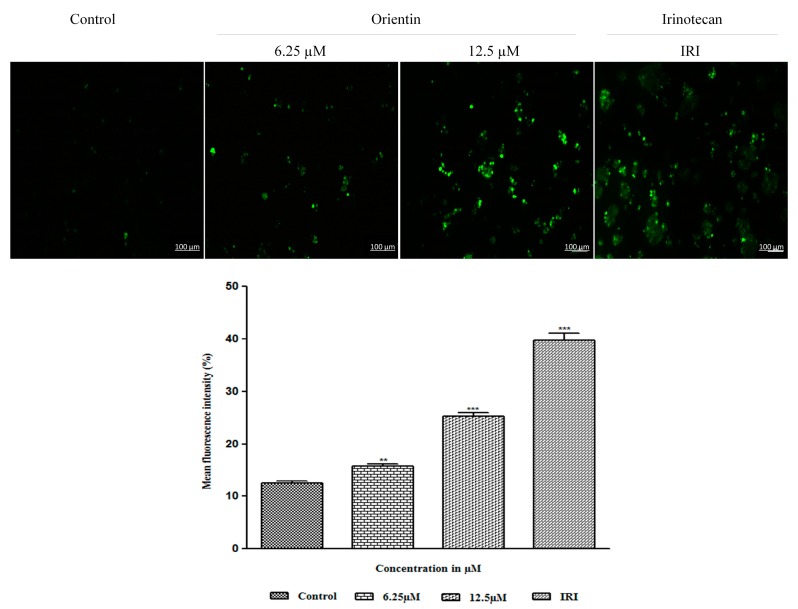
Intracellular reactive oxygen species (ROS) accumulation in HT29 cells by orientin. The fluorescent microscopic images of intracellular ROS generated in HT-29 cells treated with different concentrations of orientin and standard irinotecan for 24 h. The fluorescent images were taken with a fluorescence microscope and analyzed with Image J software. Data are shown as mean ± SD of three parallel independent experiments. ** *p* < 0.01 and *** *p* < 0.001 vs. control.

The enhancement of oxidative stress by triggering intracellular ROS generation could be a potential therapeutic strategy for treating colon cancer. Although, ROS are involved in multiple signaling cascades of tumor development, its excessive generation could lead to causing severe harm to DNA and proteins, leading to apoptosis [23]. To verify whether orientin induces apoptosis by triggering ROS generation in HT29 cells, treated cells were stained with CM-H2DCFDA, a non-fluorescent derivative of 2,7-dichlorofluorescein. Orientin significantly increased (** *p* < 0.01 and *** *p* < 0.001) intracellular ROS levels in HT29 cells in a dose dependent manner as revealed by the increase in intensity (Figure 7). The increased intracellular accumulation of ROS in treated cells was observed at both 6.25 µM and 12.5 µM similar to that of irinotecan.

### 3.7. Orientin Modulates Bcl-2 Family Proteins

**Figure 8:**
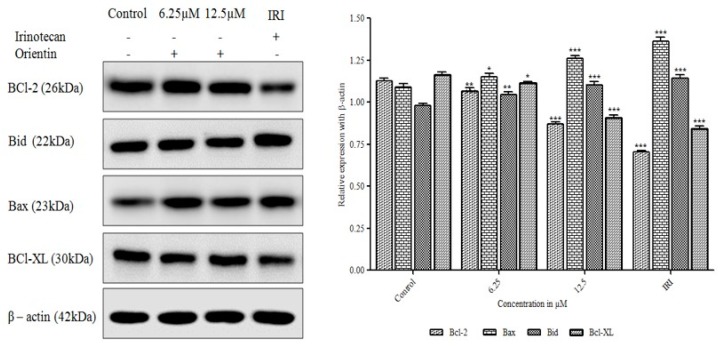
Effect of orientin on expression of Bcl-2 family proteins. HT-29 cells were treated with orientin (6.25 and 12.5 μM) and irinotecan for 24 h, respectively. Orientin increases proapoptotic Bax and Bid proteins and decreases the expression of anti-apoptotic Bcl-2 and Bcl-XL proteins. β-actin was used as an internal control. Quantitative expression of proteins shown after normalization to β-actin. Data are represented as the mean ± SD of three independent experiments (* *p* < 0.05, ** *p* < 0.01 and *** *p* < 0.001 vs. control).

The mechanisms underlying orientin-induced apoptosis in HT29 cells was ascertained by the western blot analysis of apoptosis-related proteins. After being treated with 6.25 and 12.5 µM orientin, the expression of Bcl-2 and Bcl-XL proteins were significantly reduced in a concentration dependent manner (* *p* < 0.05, ** *p* < 0.01 and *** *p* < 0.001, respectively). The proapoptotic Bax and Bid was significantly increased in HT29 cells in a concentration dependent manner (** *p* < 0.01 and *** *p* < 0.001) when compared to control. As shown in Figure 8, the reduced Bcl-XL expression accompanied by increased expression of Bax and Bid illustrates the loss of mitochondrial membrane potential in orientin treated HT29 cells.

### 3.8. Cytochrome C Release and Translocation of Smac/DIABLO by Orientin

**Figure 9:**
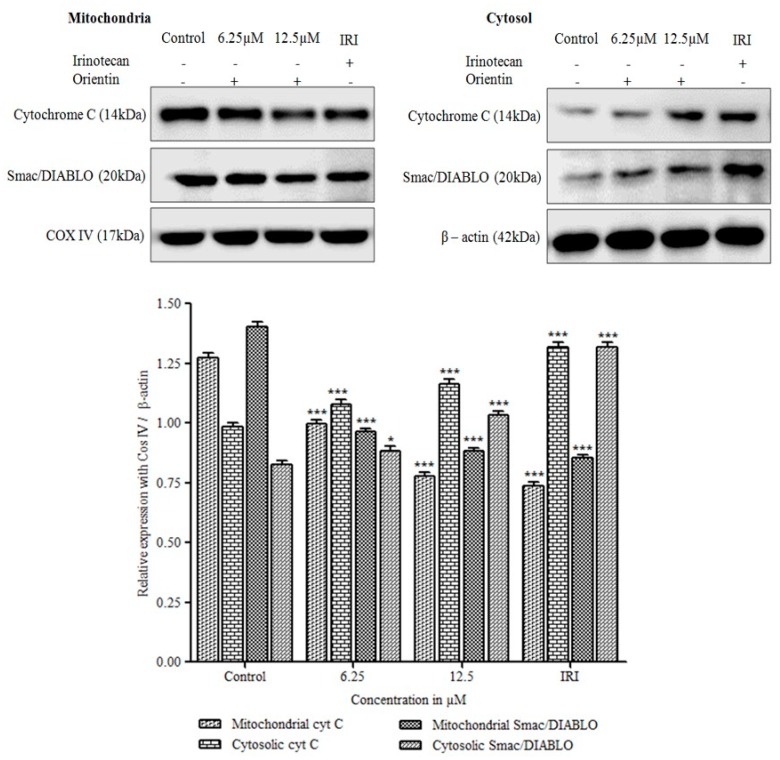
Effect of orientin on cytochrome C release and Smac/DIABLO protein. Orientin induces the translocation of cytochrome C and Smac/DIABLO from mitochondria to cytosol in HT29 cells. β-actin was used as an internal control. Quantitative expression of proteins has shown after normalization to β-actin. Values are represented as the mean ± SD of three independent experiments (* *p* < 0.05, ** *p* < 0.01 and *** *p* < 0.001 vs. control).

As shown in Figure 9, orientin significantly induced the release of mitochondrial cytochrome C to cytosol in a concentration dependent manner (*** *p* < 0.001). The mitochondrial cytochrome C was reduced significantly when compared with untreated cells (*** *p* < 0.001), in contrast, cytosolic cytochrome C was gradually increased with an increase in concentration. The pro-apoptotic mitochondria derived Smac/DIABLO protein was also found to be translocated from mitochondria to cytosol significantly (*** *p* < 0.001). Our results, demonstrated that orientin induced disintegration of mitochondrial transmembrane potential in HT29 cells and pore formation might be due to the dimerization or activation of pro-apoptotic proteins thereby accompanying the release of caspases that were prominently responsible for apoptosis.

### 3.9. Orientin Activates Caspase Cascade and Induces PARP Cleavage

Apoptosis can be either intrinsic or extrinsic depending upon the death receptors activation or the release of mitochondrial membrane potential regulatory proteins. Cytochrome C release and increased cytosolic Smac/DIABLO indicates the caspase cascade activation and inhibition of apoptotic proteins. To determine the pathway of orientin triggered apoptosis, the immunoblot analysis of caspase-3 (initiator caspases), caspase-9 (effector caspases) and their cleaved forms were carried out.

**Figure 10:**
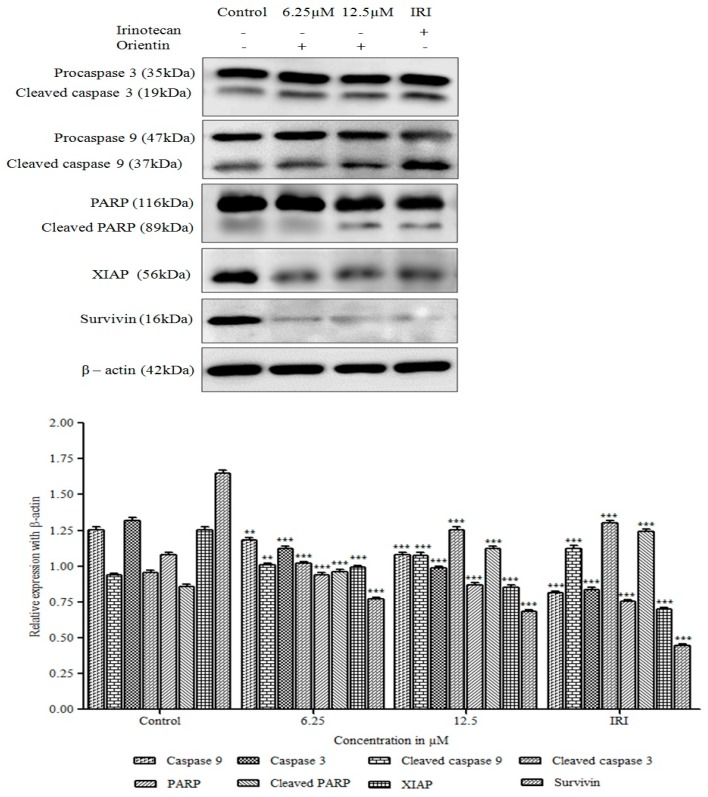
Effect of orientin on caspase cascade and PARP cleavage. HT29 cells treated with orientin activates caspase cascade and subsequently induces PARP cleavage. A marked reduction was observed in the expression of X-linked inhibitor of apoptotic proteins (XIAP) and survivin after treatment with orientin. β-actin was used as an internal control. Quantitative expression of proteins has shown after normalization to β-actin. Values are represented as the mean ± SD of three independent experiments (* *p* < 0.05, ** *p* < 0.01 and *** *p* < 0.001 vs. control).

As shown in Figure 10, caspase-3 and caspase-9 were found to be increased dose dependently in orientin treated cells (* *p* < 0.05 and ** *p* < 0.001), apparently, cleaved caspase-3 and caspase-9 were also found to be increased gradually with increasing concentration. Further, examined the effect of orientin on PARP cleavage, the hallmark of apoptosis. The expression of cleaved PARP increased significantly (* *p* < 0.05 and ** *p* < 0.001) in a concentration dependent manner after orientin treatment. These results suggested that orientin induced intrinsic apoptosis via caspase activation and cleavage of PARP.

### 3.10. Orientin Blocks the Inhibitor of Apoptotic Proteins (IAP)

**Figure 11:**
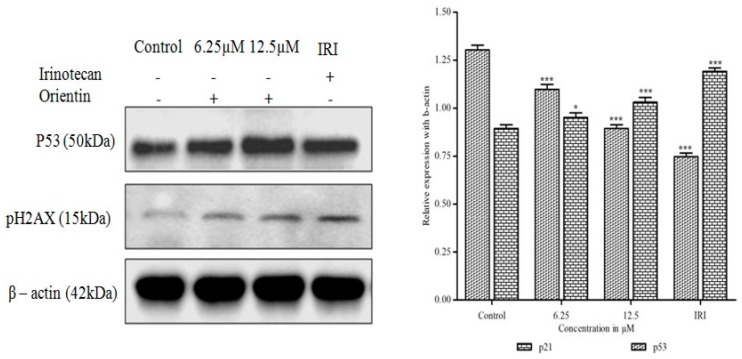
Effect of orientin on p53 and DNA damage. HT29 cells have shown a decrease in the expression of p53 and increase in the expression of pH2AX protein involved in DNA repair. β-actin was used as an internal control. Quantitative expression of proteins has shown after normalization to β-actin. Values are represented as the mean ± SD of three independent experiments (* *p* < 0.05, ** *p* < 0.01 and *** *p* < 0.001 vs. control).

To determine the effect of orientin on inhibitor of apoptotic proteins (IAP), the analysis of expression of X-linked (X) IAP and survivin, which regulates apoptosis by the inhibition of caspases, were performed. A marked reduction was observed in the expression of XIAP and survivin after treatment with orientin (Figure 11).

These results suggested that orientin induced apoptosis by blocking the IAP and thereby activating mitochondria mediated caspase dependent intrinsic apoptosis.

### 3.11. Orientin Induces p53 Expression and DNA Damage

To examine whether orientin could inhibit p53 activation and DNA damage, the expression of p53 and γ-H2AX were assessed by western blotting analysis. The tumor suppressor, p53, was found to be significantly increased after treatment with orientin in a dose dependent manner (* *p* < 0.05 and ** *p* < 0.001) as shown in Figure 11. The increased expression of tumor suppressor p53 after treatment with orientin suggests that p53 could induce the over expression of p21^WAF1/CIP1^, which further inhibited cyclin-CDK complexes activation. The increased level of γ-H2AX with increasing concentration serves as a hallmark of DNA damage after treating with orientin confirmed the DNA damage in HT29 cells.

## 4. Discussion

Orientin has been reported to exhibit potent anti-proliferative effect against EC109 and MCF-7 cells [24], however, the antiproliferative mechanism is not precisely understood. In this regard, investigated the inhibitory effect of orientin on HT29 cells and the possible molecular mechanism was explicated. The tetrazolium based antiproliferative study revealed that orientin significantly inhibited HT29 cell viability in a dose dependent manner. The light microscopic observations showed typical variations in cell morphology after 24 h exposure. This included the cell shrinkage from its polyhedral origin, rounded off, membrane blebbing and detachment of cells making substantiation for apoptosis [19]. The delimited cell cycle progression and evasion of apoptosis are the common events in the development of colon cancer [25]. Song et al. [26] suggested that the induction of cell cycle arrest at a specific checkpoint and initiation of apoptosis are often used mechanisms for treating cancer with cytotoxic agents. Cell cycle checkpoints fortify the dividing cells from DNA damage and ensure the maintenance of genomic integrity. The increased proportion of G0/G1 cells after treatment illustrates that orientin induced G0/G1 arrest in a dose dependent manner. G0/G1 arrest suggests that the initiation of cell cycle arrest may be accountable for the anti-proliferative potential of orientin.

Cell cycle is firmly driven by activated cyclin dependent serine/threonine kinases and their regulatory cyclin subunits. These cyclin/CDK complexes serve to be a biomarker for proliferation and attractive therapeutic targets for development of anticancer drugs [27]. Cyclin D and cyclin E, together with CDK2 and CDK4 regulate mitotic division and progress the cell cycle through G1 phase. Our results showed that orientin notably decreased the expression of cyclin D1, cyclin E, CDK2 and CDK4. This is in corroboration with our earlier findings where pelargonidin exhibited such a reduction in the expression of CDKs and cyclins in HT29 cells [21]. p21^WAF1/CIP1^, the major inhibitor of cyclin D/CDK complex was observed to be elevated dose dependently. The increased level of p21^WAF1/CIP1^ suggesting that orientin induced p21^WAF1/CIP1^ mediated G0/G1phase arrest in colon HT29 cells, which is in corroboration with earlier findings [28]. Phosphorylation of Rb by CDK4 initiates an intricate process of phosphorylation-mediated disruption of tumor suppressor function that releases E2F and instigates subsequent G1 to S phase progression [29]. Our experimental results indicated that orientin decreased pRb expression and thereby inhibited the subsequent progression of the cell cycle. The enhancement of oxidative stress by triggering intracellular ROS generation could also be a potential therapeutic strategy for treating colon cancer. Although, ROS are involved in multiple signaling cascades of tumor development, its excessive generation could lead to causing severe harm to DNA and proteins, leading to apoptosis [23]. Our experimental findings suggest that, orientin triggered intracellular ROS production extensively in a concentration dependent manner, which could result in the loss of mitochondrial membrane potential and intrinsic apoptosis.

Normally, phosphatidyl serine predominantly exists on the cytosolic side of the membrane, whereas during apoptosis, it gets translocated towards the outer side of the membrane. Such translocation results in the loss of plasma membrane asymmetry, an early marker for apoptosis. Annexin V, the extracellular Ca^2+^ dependent phospholipid binding protein binds to phosphatidyl serine on the cell surface and the permeability of PI in apoptotic cells detects the heterogeneous distribution of cells in early, late apoptotic phases and dead cells [30]. Annexin V-FITC/PI staining revealed that orientin treated HT29 cells significantly underwent apoptosis compared to untreated cells (*p* < 0.05) and the percentage of both early and late apoptotic cells also significantly increased in a concentration dependent manner. Thus, Annexin V-FITC/PI double staining of HT29 cells treated with orientin substantiates the apoptotic effect of orientin. Proteins of Bcl-2 family play a significant part in apoptosis and targeting those proteins have become an effective strategy for treating cancer. These intracellular proteins control pro-apoptotic, anti-apoptotic signals and mediate the membrane potential of mitochondria [24]. Bax induces apoptosis by the loss of mitochondrial permeability in response to cellular stresses. In contrast, Bcl-2 hinders cell death by inhibiting Bax [31]. The decreased level of anti-apoptotic Bcl-2 and Bcl-XL expression accompanied by the increased pro-apoptotic Bax and Bid levels after being treated with orientin demonstrates the significant therapeutic potential of orientin in modulating Bcl-2 family proteins and thereby inducing apoptosis by disruption of outer mitochondrial membrane in HT29 cells.

**Figure 12:**
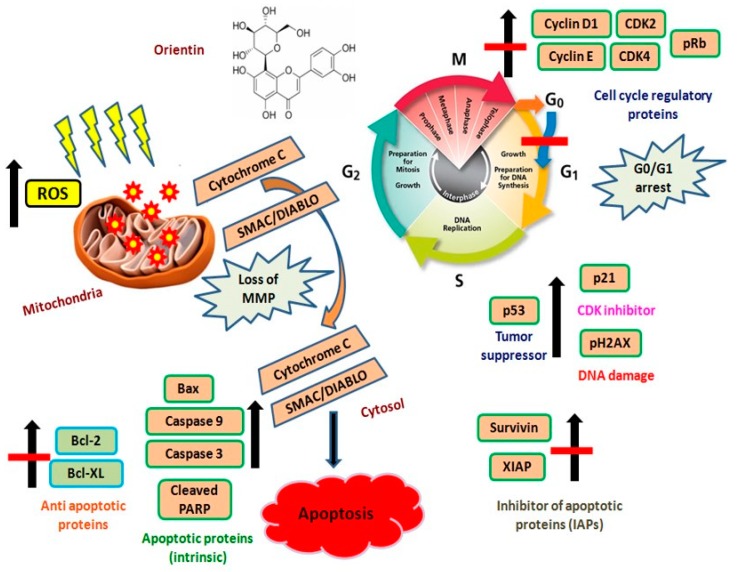
Schematic representation of orientin triggered ROS mediated mitochondrial mediated intrinsic apoptosis.

Cytochrome C release from mitochondria to the cytosol occurs during the early apoptosis. Multiple evidences suggest that active Bax protein ensures apoptosis via pore formation in mitochondrial outer membrane leads to cytochrome C release [32,33,34]. Orientin activates Bax and translocates cytochrome C to cytosol. Our results are consistent with previous findings where the pro-apoptotic protein Bid mediated Bax activation, which in turn facilitates cytochrome C release and mitochondrial membrane permeabilization [21]. Orientin induces the release of Smac/DIABLO, a mitochondrial membrane regulatory protein along with cytochrome C. This may promote a cytochrome C-dependent caspase cascade by neutralizing IAP [35,36,37]. The permeabilization of mitochondria and the cytochrome C release substantiates that orientin may induce apoptosis through intrinsic pathway. The cytosolic cytochrome C interacts with apoptotic protease activating factors (Apaf-1) and pro caspase-9 to make apoptosome and initiates the activation cascade of caspases. The binding of Apaf-1 induces conformational change in pro caspase-9 to its active proteolytic caspase-9 [38]. After treatment, an obvious increase in caspases (caspase -9 and caspase-3) activity and cleavage of PARP, an early marker of chemotherapy induced apoptosis, suggesting that orientin induces apoptosis mainly in the intrinsic pathway [39,40]. The decrease in the expression of IAP family members, XIAP and survivin may be attributed to the release of cytochrome C and the depolarization of mitochondrial membrane to elicit the activation of the caspase cascade by orientin [37]. The increased expression in tumor suppressor p53 after treatment with orientin suggests that p53 could induce the over expression of p21^WAF1/CIP1^, which inhibits the cyclin-CDK complexes. The increased level of γ-H2AX serves as a hallmark of DNA damage after treating with orientin confirms the DNA damage induced in HT29 cells. The overall schematic representation of orientin triggered ROS mediated mitochondrial mediated intrinsic apoptosis was shown in Figure 12.

## 5. Conclusions

The study demonstrated the anti-proliferative potential of dietary C-glycosyl flavonoid orientin against human CRC HT29 cells by rendering cytotoxicity, morphological changes, chromatin condensation, membrane blebbing and nuclear fragmentation. The overall findings deliberately state that orientin arrested the cell cycle in the G0/G1 phase, induced intracellular ROS and simultaneously depolarized the mitochondrial membrane to activate caspase dependent intrinsic apoptosis. Therefore, the dietary orientin rich in rooibos tea might be a promising chemotherapeutic agent against CRC in human.
